# Pore Forming Properties of Cecropin-Melittin Hybrid Peptide in a Natural Membrane

**DOI:** 10.3390/molecules14125179

**Published:** 2009-12-11

**Authors:** Alberto Milani, Mascia Benedusi, Marco Aquila, Giorgio Rispoli

**Affiliations:** Dipartimento di Biologia ed Evoluzione, Sezione di Fisiologia e Biofisica, National Institute of Neuroscience and Neuroscience Center, Università di Ferrara, Via L. Borsari 46, I-44100 Ferrara, Italy; E-Mails: alberto.milani@student.unife.it (A.M.); bndmsc@unife.it (M.B.); marco.aquila@student.unife.it (M.A.)

**Keywords:** peptide antibiotics, pore-forming toxins, photoreceptors, ion channels, patch clamp

## Abstract

The pore forming properties of synthetic cecropin-melittin hybrid peptide (Acetyl-KWKLFKKIGAVLKVL-CONH_2_; **CM15**) were investigated by using photoreceptor rod outer segments (OS) isolated from frog retinae obtained by using the whole-cell configuration of the patch-clamp technique. **CM15** was applied (and removed) to (from) the OS in ~50 ms with a computer-controlled microperfusion system. Once the main OS endogenous conductance was blocked with light, the OS membrane resistance was ≥1 GΩ, allowing high resolution, low-noise recordings. Different to alamethicines, **CM15** produced voltage-independent membrane permeabilisation, repetitive peptide application caused a progressive permeabilisation increase, and no single-channel events were detected at low peptide concentrations. Collectively, these results indicate a toroidal mechanism of pore formation by **CM15**.

## 1. Introduction 

**Figure 1:**
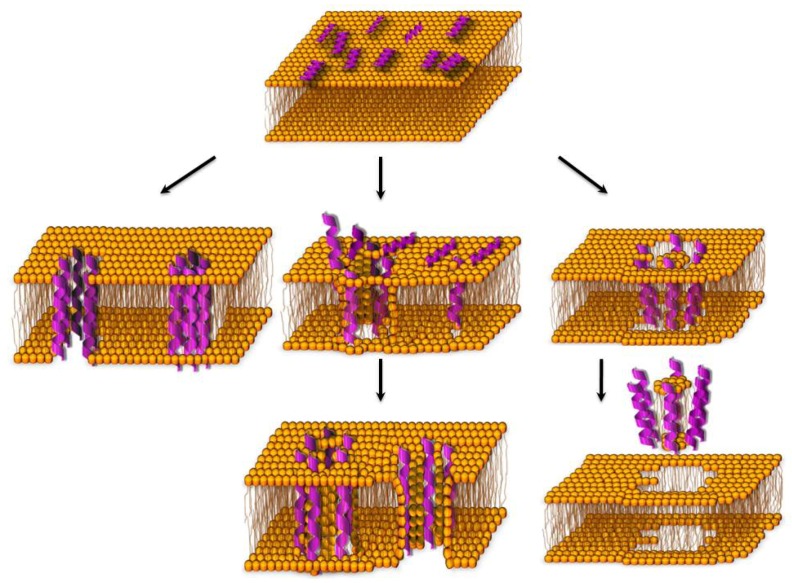
Mechanisms of cell membrane permeabilisation induced by a peptide. After adhering on the external face of the membrane (top), the peptide could insert in the membrane according to a barrel-stave (left), toroidal (two consecutive center diagrams), or carpet mechanism (right diagrams).

The antimicrobial peptides form an essential part of the innate immunity against pathogens; many peptides induce bacterial death by permeabilising their plasma membrane to the point of inducing cell lyses. This permeabilisation occurs following the adhesion of the peptides parallel to the bacterial lipid bilayer, the peptide orientation perpendicular to the membrane, and finally to their insertion into the bilayer, forming transmembrane pores. Because of their amino-acid composition, amphipathicity, and cationic charge, three distinct mechanisms have been proposed to explain membrane permeabilisation: “barrel-stave”, “carpet”, and “toroidal-pore" (Figure 1; reviewed in [1]). 

Briefly, the barrel-stave mechanism requires that peptides bind together (as staves) around a central lumen, forming a pore (the barrel); the peptide hydrophobic segments align with the lipid core region of the bilayer, while their hydrophilic segments face the lumen interior. In the toroidal-pore, the polar segments of the peptides associate with the polar head groups of the lipids so that the lipids are forced to tilt from the lamellar normal up to connect the two leaflets of the membrane, forming a continuous bend from one side to the other of the membrane, in the fashion of a toroidal hole. Differently from the barrel-stave mechanism, the peptides are always associated with the lipid head groups, even when they are perpendicularly inserted in the lipid bilayer. In the carpet mechanism, the peptides accumulated on the bilayer surface are attracted by Coulomb forces to the anionic phospholipid head groups at several sites, covering the membrane surface in a carpet-like manner. The peptides then assemble with the lipids to form transiently toroidal pores, allowing more and more peptides to access the membrane. Eventually, the surface-oriented peptides lead to the bilayer disintegration in a detergent-like manner, by forming micelles. It is, however, conceivable that these three mechanisms of membrane permeabilisation are not completely different and independent: for instance, it is possible that a certain peptide may act preferentially with one mechanism at a certain concentration and with another mechanism at a higher concentration [1], or in the presence of some other endogenous or exogenous molecules. 

**Figure 2:**
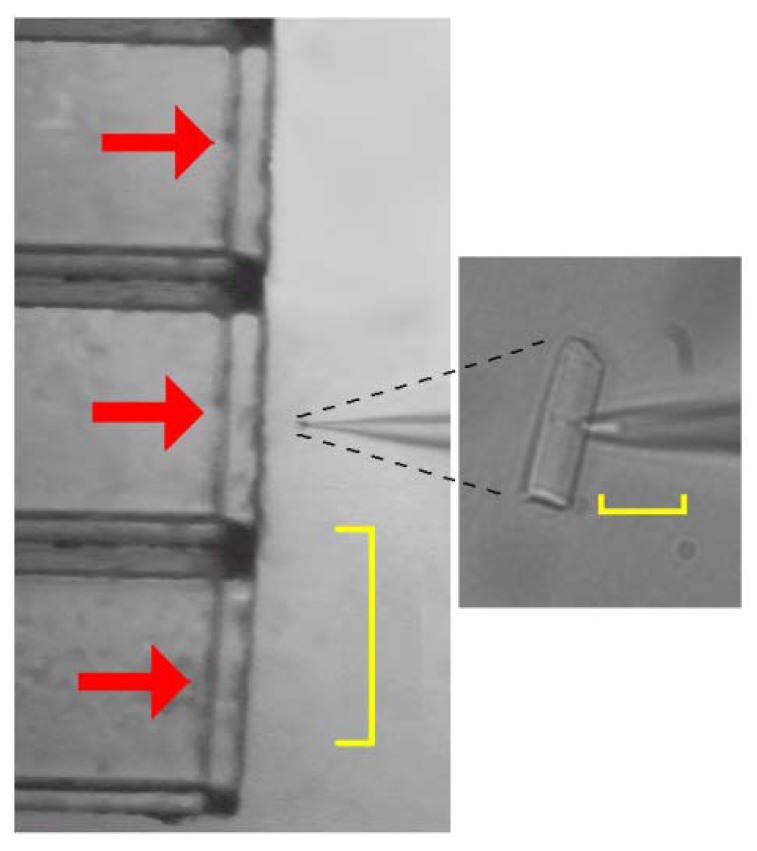
The technique employed to investigate the permeabilisation properties of the peptides inserted in a natural membrane. *Left* panel, isolated rod outer segment (OS) recorded in whole-cell mode (visible on the right-side of this microphotograph) aligned in front of the multibarreled perfusion pipette (visible on the left-side; scale bar is 500 µm; horizontal red arrows denote perfusion flows); *Right* panel, the same OS at higher magnification (scale bar is 20 µm).

A small 15-residue synthetic hybrid peptide (**CM15**; see Experimental Section), first described by [2], composed of the first seven residues of the silk moth cecropin A and residues 2-9 from the bee venom peptide melittin, displays potent broad-spectrum antimicrobial activity, but seems to lack the haemolytic properties associated with melittin. It is not clear whether **CM15** forms discrete membrane pores or disrupts membranes via a detergent-like carpet mechanism [2,3,4,5]. In this paper, a novel strategy has been employed to precisely assess the biophysical characteristics and the pore formation dynamics of **CM15** under physiological conditions. Briefly, a custom-made, computer controlled microperfusion system was employed to rapidly apply (and remove) natural and custom-made peptides onto a cell, recorded in voltage-clamp, whole-cell configuration, in which all the endogenous conductances were fully blocked [6]. Peptide insertion dynamics was therefore given by the time course of the exogenous current at a given potential (*V_h_*). It has been found that the isolated rod outer segment (OS) [7] was particularly suitable to carry on the above studies, because it was possible to easily block all the endogenous currents without using any drug (such as tetrodotoxin, tetraethylammonium, dihydropyridines, *etc*.), that could aspecifically interfere with the pore formed by the peptides as well. The computer controlled microperfusion system, developed in collaboration with an industrial partner (De Angelis s.r.l., Genoa, Italy; Figure 2), allowed us to apply and remove ions, drugs and peptides on the cells in ~50 ms with an accurately controlled timing. This allowed to study, besides the ion channel characteristics (such as the presence of gating, its voltage dependence, the unitary conductance, and the selectivity and blockade [8]), the dynamics of pore formation as well, that are of key importance to assess the channel performance and its potential biotherapeutic activity.

## 2. Results and Discussion 

To study the biophysical properties of a pore-forming peptide inserted in a natural membrane, it was planned to extracellularly perfuse the peptides on a cell where all the endogenous sources of membrane current could be readily blocked. The photoreceptor rod outer segments (OS) mechanically isolated from *Rana esculenta* have been found particularly suitable to carry on this study, because of their large size (Figure 2, *Right* panel) and for the commercial availability and low cost of this frog species. The vertebrate OS possesses just two endogenous conductances: the light sensitive channels and the Na^+^:Ca^2+^, K^+^ exchanger (reviewed in [9,10]). If the OS is illuminated, the light sensitive channels close; moreover, the exchanger can be blocked if just one of the ion species transported by it (*i.e*., Na^+^, Ca^2+^ or K^+^) is removed from both sides of the membrane [11,12].

**Figure 3:**
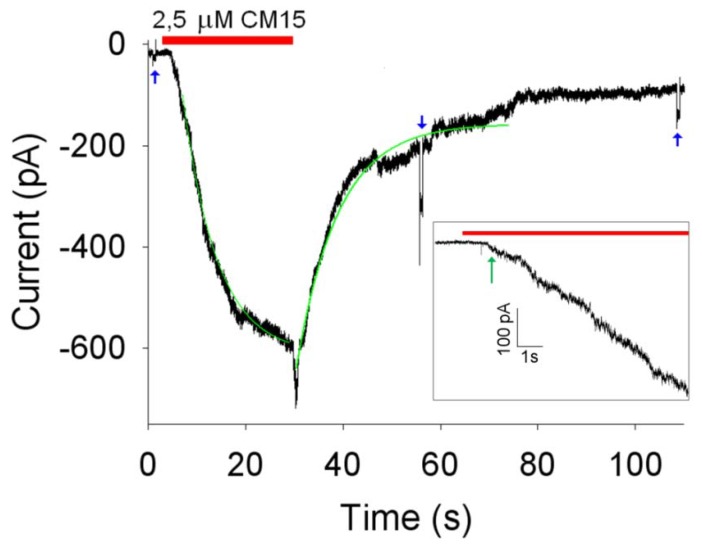
Kinetics of OS membrane permeabilisation induced by 2.5 μM concentration of **CM15**. Red bar indicate the timing of the peptide application and withdrawal; green traces are exponential fittings of the activation and deactivation phases of the current (*τ_a_* ≈ 7 s; *τ_d_* ≈ 8.5 s; *I_max_* ≈ -580 pA; *V_h_* = -20 mV); blue arrows are 700 ms voltage steps of -10 mV superimposed to *V_h_* to measure *R_m_* (that was 1 GΩ before peptide application and 200 MΩ within 50 s after peptide removal from the external perfusion solution). *Inset*: initial activation phase of the current at high resolution; red bar indicate the timing of peptide application (*D_a_* ≈ 1.0 s, indicated by the green arrow; *D_d_* ≈ 0.5 s).

**Figure 4:**
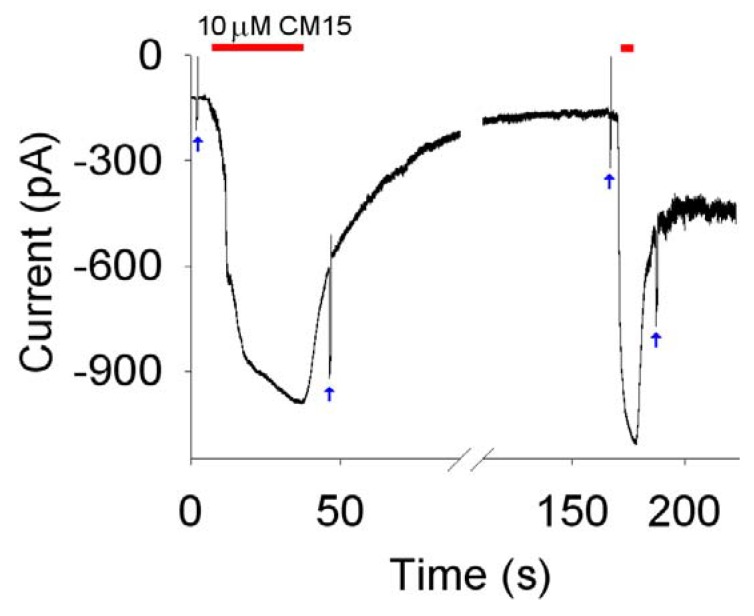
Kinetics of OS membrane permeabilisation induced by 10 μM concentration of **CM15**. Red bars indicate peptide application and withdrawal timing; blue arrows indicate the -10 mV steps used to measure *R_m_* before (~200 MΩ) and at the steady state after peptide removal (~120 MΩ following the first application and ~45 MΩ following the second one). Kinetics parameter relative to the second application (where the current was maximal): *D_a_* ≈ 0.6 s; *D_d_* ≈ 0.4 s; *τ_a_* ≈ 1.1 s; *τ_d_* ≈ 2.7 s; *I_max_* ≈ -930 pA.

**Figure 5:**
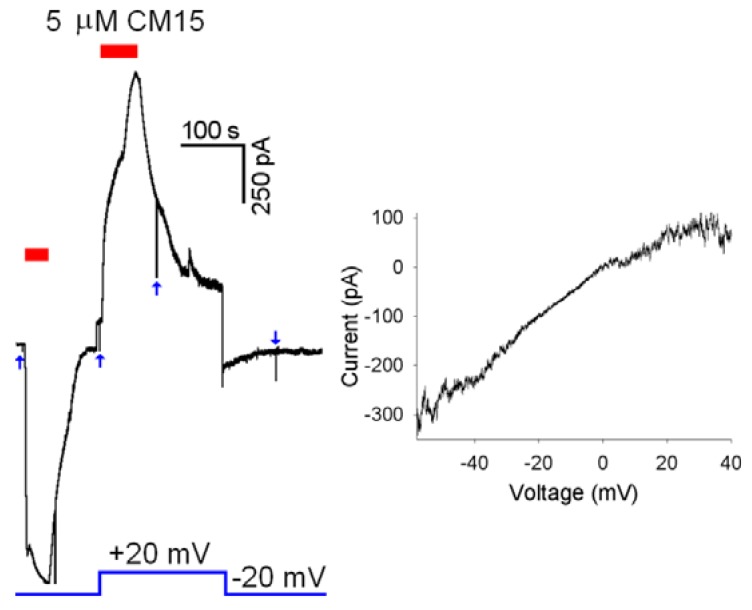
Voltage dependency of membrane permeabilisation induced by **CM15**. *Left* panel, 5 µM of **CM15** applied at *V_h_*=-20 mV and +20 mV; *Right* panel, current to voltage relationship obtained using a voltage ramp on a different cell (see text) when current attained a plateau (-97 pA at *V_h_*=-20 mV) during perfusion with 2.5 µM of **CM15**. Blue arrows indicate the -10 mV steps used to measure *R_m_*.

To simplify the interpretation of the experiments, patch pipettes were filled with the same perfusion solution (that typically contained 130 mM of KCl) to ensure the current was only driven by the holding potential (*V_h_*, usually set to –20 mV). Under these ionic conditions and under room lights (that will close all the light-regulated channels), the OS membrane resistance (*R_m_*) was usually larger than 1 GΩ in the absence of the peptide, exhibiting a linear (ohmic) current-to-voltage characteristics [6]. This high *R_m_* value allowed current recordings with a resolution of 1 pA in a bandwidth of at least 1 kHz; however, to preserve the membrane integrity during long recordings, it was necessary to include a physiological concentration of Ca^2+^ (1 mM) to the external solution. Therefore, 1 mM Ca^2+^ was added to the intracellular solution as well, to ensure that the current was still entirely driven by *V_h_*. The dynamics of the pore formation was tested by means of the following protocol (Figure 3). With the isolated OS continuously held to *V_h_*, *R_m_* was measured before peptide perfusion by means of a brief -10 mV step; the peptide was then quickly applied (in about ~50 ms) using the fast perfusion system. Once the current had stabilised, the OS was finally returned to the control solution (without the peptide) to assess a possible recovery of the current, and *R_m_* was again measured. Concentrations of CM 15 ≤ 1 µM gave no detectable macroscopic currents nor single channel events, in contrast to alamethicin [6], that generated clear single channel current at concentrations ≤250 nM. Macroscopic currents were routinely obtained at **CM15** concentrations ≥2.5 µM; repetitive peptide applications produced currents of increasingly amplitude (Figure 4 and Figure 5). These observations exclude the fact that **CM15** permeabilises the plasma membrane according to a barrel-stave mechanism. The latter requires that a certain number of monomers binds together once in the plasma membrane to form an ion conductive pore: if the peptide concentration is small, then pores are formed and disaggregated frequently, producing single channel events; at higher concentrations, membrane peptides equilibrate with the ones externally perfused, giving a stable macroscopic current [6,13]. The increase in plasma membrane permeability to ions induced by **CM15** can be described quantitatively by the following kinetic parameters:

The activation delay (*D_d_*), defined as the time lag between peptide application and the time in which the current deviates from its baseline (following peptide application), more than three times the noise average fluctuation (indicated by the green arrow in the Figure 3, *Inset*);

The activation time constant (*τ_a_*), defined as the time constant of the single exponential fit to current activation (Figure 3);

The current amplitude at steady-state (*I_max_*);

The deactivation delay (*D_d_*), defined as the time lag between peptide removal and the time in which the current deviates from its baseline more than three times the noise average fluctuation;

The deactivation time constant (*τ_d_*) defined as the time constant of the single exponential fit to current deactivation (Figure 3).

However, these parameters cannot be unambiguously estimated, since they may change as the current increases (compare, for instance, *τ_a_* at 2.5 and 10 µM of **CM15** reported in the captions of Figure 3 and Figure 4). To obtain a reproducible value of these parameters, recordings were selected to approximately give current amplitudes between 400 and 600 pA, irrespective of the peptide concentration. A representative example of one of these recordings is shown in Figure 3; the average value of the kinetics parameters were: *D_a_* ≈ 0.8 ± 0.2 s; *D_d_* ≈ 0.6 ± 0.2 s; *τ_a_* ≈ 8.4 ±1.4 s; *τ_d_* ≈ 10 ± 2 s; *I_max_* ≈ -510 ± 51 pA (*V_h_* = -20 mV; *n* = 6). The largest values of the kinetics parameter were measured by using the **CM15** at 10 µM concentration; larger concentrations were not used to avoid the loss of voltage control. Indeed, as the current increases, there is a voltage error induced by the access resistance (that was typically ~15 MΩ in the recordings considered in this paper) that can be as high as 15 mV at 1 nA of current.

In contrast to alamethicin, the current did not return to the zero level following peptide removal [6,13]: the current recovered instead to a plateau level and the *R_m_* measured on this plateau was consequently smaller in respect to the one measured before the **CM15** application. The **CM15** concentration was larger, and/or more and more applications were performed (*i.e*., the larger was the current induced by **CM15**), the larger was the plateau amplitude and the smaller was the *R_m_* (measured during the plateau phase). The incomplete recovery in respect to alamethicin and the progressive current increase observed with repetitive **CM15** applications indicate that **CM15** makes pores that are relatively stable and that are larger and larger and/or more and more numerous with repetitive applications. However, the substantial recovery of current and the cell integrity, observed even at high **CM15** concentrations (Figure 4), exclude the carpet mechanism of membrane permeabilisation: micellation is expected to be severe under these conditions, producing the irreversible disruption of the membrane and cell lyses. Therefore, it can be concluded that **CM15** permeabilises the membrane according to a toroidal mechanism of pore formation. This view is also supported by the voltage-independency of **CM15** membrane permeabilisation, in contrast to alamethicin, that instead inserts in the OS membrane at negative voltages [6,13]. Indeed, **CM15** application at +20 mV or -20 mV produced currents with similar *I_max_* (and *D_a_*, *D_d_*, *τ_a_*, *τ_d_* as well; Figure 5, *Left* panel). However, the latter protocol is not suitable to assess the precise voltage dependency of the current, since the current progressively increases with repetitive application of **CM15** (note that the maximal current amplitude at +20mV in Figure 5 is larger in respect to the one recorded at -20 mV). To circumvent this problem, rapid voltage ramps (slope: 0.25 mV/ms) were applied during **CM15** perfusion at *V_h_* =-20 mV, waiting for the current to stabilise for a period at least as long as the ramp (400 ms). To avoid the loss of voltage control at extreme voltages (-60 and +40 mV; see above), cells were selected to have currents of about 100-200 pA at *V_h_* =-20 mV. The resulting current to voltage relationship was almost perfectly ohmic for physiological voltages (Figure 5, *Right* panel) in all cells examined (*n* = 4).

Remarkably, the aminoacids of **CM15** must be lined up in a precise sequence to produce efficient membrane permeabilisation: indeed, a random sequence of these aminoacids, like the one reported in the Experimental section (scrambled **CM15**), is not able to produce any permeabilisation, even for repetitive applications at 10 µM concentration (lasting up to 3 min; *n* = 3 OS; data not shown).

## 3. Experimental Section

Rod outer segments (OS) were mechanically isolated from the retina of the *Rana esculenta*. Methods are described in detail elsewhere [6]. Animal experiments and care were performed in compliance with the Declaration of Helsinki guidelines and a local ethical Committee approved the experimental procedures. All manipulations were made in the dark using infrared illumination and an infrared viewer (Find-R-Scope, FJW Optical Systems, Palatine, IL, USA). Before dissection, the animal was dark adapted (≈4 hr), anaesthetized by immersion in a tricaine methanesulphonate solution (1 g/L in water), and then decapitated. Both eyes were removed from the head and hemisected. The back half of the eyeball was cut into pieces (up to four) that were stored in oxygenated Ringer solution on ice and used when needed. The retina was "peeled" from an eyecup piece and was gently triturated in Ringer (~5 mL), using a fire-polished Pasteur pipette to obtain the OS. A fluid drop containing the OS was then transferred to the recording chamber. The OS on the microscope stage (TE 300, Nikon, Tokyo, Japan) were illuminated with an ultrabright infrared LED (900 nm) and viewed in a window of AquaCosmos software package (version 2.5.3.0; Hamamatsu Photonics, Tokyo, Japan), controlling via a PCI board (PCDIG, Dalsa, Waterloo, ON, Canada) a fast digital camera (C6790-81, Hamamatsu Photonics) coupled to the microscope.

OS were recorded using the whole-cell configuration of the patch-clamp technique under visual control at room temperature (20-22 °C; Figure 2). The Ringer solution had the following composition (in mM): 115 NaCl, 3 KCl, 10 HEPES free acid [*N*-(2-hydroxyethyl)piperazine-*N’*-(2-ethanesulfonic acid)], 0.6 MgCl_2_, 0.6 MgSO_4_, 1.5 CaCl_2_, 10 glucose (osmolality 260 mOsm/Kg, buffered to pH = 7.6). All chemicals were purchased from Sigma (St. Louis, MO, USA).

The current amplitude (recorded employing an Axopatch 200B; Molecular Devices, Sunnyvale, CA, USA) elicited by a -10 mV pulse in cell attached and during whole-cell recording was used to measure seal resistance and *R_m_*, respectively. Once the whole cell recording was obtained, the current transients elicited by -10 mV voltage pulses were used to measure access resistance and cell capacitance; the cell was then aligned in front of a multibarrelled perfusion pipette that can be moved on an horizontal plane (Figure 2, *Left* panel). Peptides were applied and removed in ~50 ms by switching forth and back the OS from a stream of control perfusion solution [composition (in mM): 130 K^+^, 1 Ca^2+^ and 10 HEPES; osmolality 260 mOsm/Kg, buffered to pH=7.6 with KOH] to a stream containing the peptide (dissolved in the same perfusion solution). This strategy allowed us to assess the dynamics of the pore formation and the possible reversibility of this process [6]. Patch pipettes were filled with the same perfusion solution in order to drive the current just with the *V_h_* (that was typically –20 mV).

The primary structure of **CM15** peptide was: acetyl-KWKLFKKIGAVLKVL-CONH_2_; as a control, to ensure that the **CM15**-induced permeabilisation was not due to some aspecific effect produced by the interaction of one or more of its aminoacids with some membrane protein, a “scrambled” version of **CM15** was tested (primary structure: acetyl-KWKLKFKIGLVKLVAV-NH_2_). Both peptides were a generous gift of Dr. Feix (of Department of Biophysics, Medical College of Wisconsin, Milwaukee, WI, USA). **CM15** and its scrambled version were dissolved in bidistilled water to get a 500 μM stock solution; an aliquot of this stock was dissolved in the perfusion solution to get a final concentration of 1, 2.5, 5 and 10 μM, and used within 30 min.

Recordings were filtered at 2 kHz via an eight-pole Butterworth filter (VBF/8 Kemo, Beckenham, UK), sampled on-line at 5 kHz by a Digidata 1322A (Molecular Devices) connected to the SCSI port of a Pentium computer running the pClamp 9.0 software package (Molecular Devices), and stored on disk. Data were further low-pass filtered off-line at 200 Hz using a Gaussian filter and analyzed using Clampfit (version 9.0; Molecular Devices). Figures and statistics were performed using SigmaPlot (version 8.0; Jandel Scientific, San Rafael, CA, USA). Results are given as means ±sem.

## 4. Conclusions 

The cecropin-melittin hybrid peptide **CM15** produced voltage-independent permeabilisation of photoreceptor rod outer segment (OS) membranes. Repetitive peptide applications at concentrations >2 µM caused the progressive increase of the steady-state current amplitude; no discernible single-channel events were detected at low peptide concentrations (*i.e.*, ≤1 µM), thus excluding a barrel-stave mechanism of membrane permeabilisation. The cell integrity and the substantial reversibility of permeabilisation observed at concentrations as high as 10 µM of **CM15** would not be expected in the case of a carpet mechanism of pore formation, because at these concentrations the micellation is expected to disrupt the OS membrane causing its lyses. Collectively, these results indicate that **CM15** inserts in the plasma membrane according to a toroidal mechanism of pore formation.
